# Pharmacy academicians’ perception towards public health: a qualitative study

**DOI:** 10.3389/ijph.2026.1608594

**Published:** 2026-09-18

**Authors:** Şeyma Halaç, Muhammed Yunus Bektay, Hasan Hüseyin Eker, Mustafa Öztürk

**Affiliations:** 1 Ministry of Health (Turkey), Ankara, Türkiye; 2 Department of Clinical Pharmacy, Faculty of Pharmacy, İstanbul University-Cerrahpaşa, İstanbul, Türkiye; 3 Department of Public Health, Hamidiye School of Medicine, University of Health Sciences, Istanbul, Türkiye

**Keywords:** Attitude of health personnel, education, pharmacy, pharmacy faculty, professional role

## Abstract

**Objectives:**

This qualitative study explored pharmacy academics’ perceptions of pharmacists’ public health roles, the adequacy of pharmacy education, and barriers to public health practice in Türkiye.

**Methods:**

Eleven pharmacy academics from Faculties of Pharmacy in Istanbul participated in online, in-depth, semi-structured interviews. Data were analysed using reflexive thematic analysis guided by the Consolidated Criteria for Reporting Qualitative Research.

**Results:**

Participants viewed public health as a multidimensional field centred on prevention and collaboration. Community pharmacies were considered accessible entry points to healthcare with substantial potential to provide preventive services, support health literacy, and contribute to chronic disease management. This potential remained underutilised due to insufficient public health competencies in pharmacy education, limited societal health literacy, and inadequate systemic recognition of pharmacists’ roles.

**Conclusion:**

Strengthening public health education in pharmacy curricula and establishing policies that recognise pharmacists as frontline public health practitioners may enhance their contribution to population health.

## Introduction

Pharmacists are among the most accessible healthcare professionals in the community [1–3]. Beyond medication supply and pharmacological counselling, community pharmacists contribute to health protection and promotion through patient education, counselling, and preventive activities [1]. Pharmaceutical public health applies pharmaceutical knowledge, skills, and resources to disease prevention and the protection and promotion of population health [4]. Internationally, these roles have become increasingly structured and visible within healthcare systems [5].

Community pharmacists may contribute to rational medicine use, treatment monitoring, immunisation, reproductive health, chronic disease management, infectious disease follow-up, healthy lifestyle counselling, cancer awareness, addiction prevention, mental health, and emergency response [2, 6–9]. Their accessibility and regular contact with the public make pharmacies suitable settings for preventive and health-promotion services.

In Türkiye, Ministry of Health Circulars No. 2019/10 and 2019/18 formally recognised community pharmacies as providers of primary healthcare services [10]. Nevertheless, evidence suggests that pharmacists’ public health potential remains underused and services are not sufficiently standardised [11, 12]. Strengthening this role requires supportive policies and stronger public health competencies through undergraduate and postgraduate pharmacy education [12, 13].

This study explored pharmacy academics’ perceptions and experiences regarding pharmacists’ public health roles, the adequacy of pharmacy education in preparing pharmacists for these roles, and barriers limiting engagement in public health activities.

## Methods

### Study design and reporting

This qualitative study used individual, in-depth, semi-structured interviews to explore pharmacy academics’ perceptions of public health and pharmacists’ public health roles. Data were analysed using reflexive thematic analysis [14–16], and reporting was guided by the Consolidated Criteria for Reporting Qualitative Research (COREQ) [17].

### Setting, participants and recruitment

Participants were pharmacy academics working at Faculties of Pharmacy in Istanbul. Purposive maximum-variation sampling sought diversity in academic rank, disciplinary background, and involvement in public health-related teaching or research. Eligibility required current employment at an Istanbul Faculty of Pharmacy, involvement in pharmacy education, at least 4 years of academic experience, and willingness to participate online. Sixteen academics were approached; 15 interviews were scheduled, and recruitment ended after 11 interviews when the research team judged that sufficient information power had been reached and substantively new codes were diminishing.

The 11 participants represented eight universities (four public and four private). Seven were female and four male; mean age was 38 years (range 29–52). Two were professors, three associate professors, and six assistant professors. Their fields were clinical pharmacy (n = 4), pharmacognosy (n = 3), pharmaceutical technology (n = 3), and pharmaceutical toxicology (n = 1). Participant characteristics are presented in Annex Table 1.

**TABLE 1:** Distribution of participants’ sociodemographic and occupational characteristics (Istanbul, Türkiye. 2024–2025).

Speaker code	University	Title	Area of expertise	Age	Gender	Duration of employment in the profession	Duration of service in academia	Involvement in processes related to pharmacy education
S1	Cerrahpaşa University	Associate professor	Pharmaceutical Technology	33	Male	12	12	Yes
S2	Medipol University	Associate professor	Pharmacognosy	35	Woman	11	11	Yes
S3	Marmara University	Professor	Clinical pharmacy	52	Male	32	31	Yes
S4	Marmara University	Professor	Clinical pharmacy	51	Woman	28	28	Yes
S5	Health Sciences University	Dr. Assistant Professor	Pharmaceutical technology	35	Male	12	12	Yes
S6	İstinye University	Dr. Assistant Professor	Pharmacognosy	33	Woman	10	8	Yes
S7	Istanbul University	Dr. Assistant Professor	Pharmaceutical technology	43	Woman	22	14	Yes
S8	Bezmialem Foundation University	Dr. Assistant Professor	Pharmaceutical toxicology	32	Woman	6	6	Yes
S9	Acıbadem University	Dr. Assistant Professor	Clinical pharmacy	39	Woman	16	6	Yes
S10	Cerrahpaşa University	Associate professor	Clinical pharmacy	37	Male	12	10	Yes
S11	Medipol University	Dr. Assistant Professor	Pharmacognosy	29	Woman	4	4	Yes

### Data collection procedures

Data were collected between November 2024 and January 2025 through Zoom or Google Meet. A semi-structured interview guide was developed from the study aim and relevant literature and policy documents. Following review by a public health academic, a pharmacy academic, and a public health specialist, and two pilot interviews, an 11-question guide was finalised. Interviews lasted approximately 40 min. With permission, interviews were audio-recorded and the researcher took notes during and after interviews.

### Transcription, research team and reflexivity

Recordings were transcribed verbatim, checked against the audio, and anonymised by removing names and institutional identifiers. Participants were assigned identifiers S1–S11; transcripts were not returned for correction. Interviews were conducted by SH, an MD and PhD candidate with qualitative interviewing and analysis experience who had a prior professional relationship with participants. Participants were informed of the study purpose. Reflexive journaling and analytic memos documented assumptions, contextual influences, and interpretive decisions.

### Data analysis and trustworthiness

Data management and coding were conducted in MAXQDA 2024 Pro. SH repeatedly read the transcripts and conducted inductive line-by-line coding. Codes were mapped and clustered into candidate domains, which the research team refined through peer-debriefing meetings. The final framework comprised 15 codes organised into four domains under one overarching theme, “The Strategic Role and Perceptions of Pharmacists in Public Health”: (i) conceptual and definitional approaches to public health; (ii) emphasis on preventive healthcare; (iii) individual and societal awareness elements; and (iv) intersections between community pharmacy and public health. Coding was led by SH, with HHE and MÖ acting as reflexive “critical friends”; disagreements were resolved through discussion focused on analytic richness rather than agreement metrics. Credibility was supported through iterative engagement, peer debriefing, and verbatim quotations, while an audit trail and reflexive journaling supported dependability and confirmability.

### Ethics and data management

The studies involving human participants were reviewed and approved by the Hamidiye Scientific Research Ethics Committee of the University of Health Sciences (approval date: September 2, 2022; decision no. 20/7; project registration no. 22/430). Participants received study information and provided written informed consent, including consent for recording. Data were anonymised and stored on a password-protected institutional hard drive accessible only to the research team. The study was conducted in accordance with the 1964 Declaration of Helsinki and its later amendments.

## Results

### Perception and definitional approaches to public health

The findings obtained within the scope of this theme reveal how the conceptual boundaries of public health are defined in the context of pharmacy practice and how individual-community approaches to this field are shaped. This theme is concentrated in the following 4 categories and their 15 sub-codes:

1. Emphasis on Preventive Healthcare
2. Individual and Community Awareness Elements
3. Conceptual and Definitional Framework
4. Points of Contact between Public Health and Community Pharmacy

The topics most intensively addressed at the code level are shown in Figure 1 in the Code Matrix.

**FIGURE 1:**
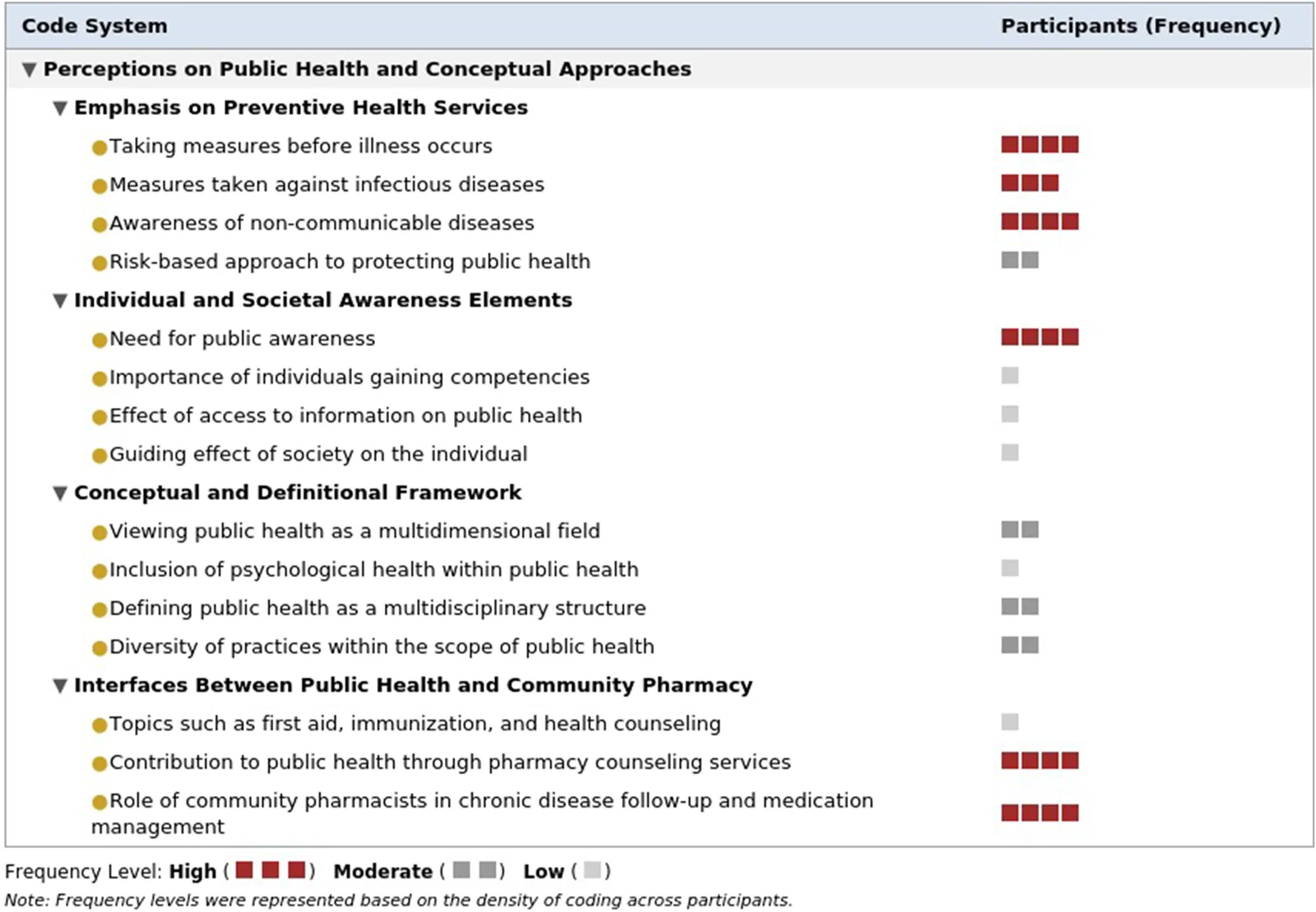
Code matrix for the theme of perceptions and definitional approaches to public health (Istanbul, Türkiye. 2024–2025).

Perceptions of public health are defined in academics’ discourses as a protective, preventive and multidimensional area. Participants see public health as a concept that is not limited to individual health but also encompasses social welfare. In this context, the code “Taking precautions before diseases occur” stands out as the most intensively discussed topic in the interviews. Academics emphasize that one of the basic functions of public health is to prevent diseases before they occur; they state that this approach has strategic importance at the individual and social level. For example, one participant expressed this view as follows:

“I think that everyone is responsible in public health. I mean, I think that physicians, pharmacists, nurses, the government, companies, everyone has their own share of responsibility. In addition, I believe that community pharmacists have a duty to public health” (S9).

This statement shows that public health is perceived as a multi-stakeholder area of responsibility and that the role of pharmacists in this process is increasingly recognized.

In addition, the code “Need to raise public awareness” is frequently included in the participants’ statements. Increasing health literacy is considered as an element that directly affects the effectiveness of public health services. Participants state that pharmacists should undertake an informative and guiding role in order to raise public awareness:

“When it comes to public health practices, health literacy actually comes to my mind. Because I think that one of the most fundamental factors in increasing public health is increasing health literacy. Unfortunately, health literacy is not at very high levels in our country” (S9).

This quote reveals that the perception that health literacy is low in Türkiye is widespread among academics and that this reduces the impact of public health services.

#### Emphasis on preventive healthcare

Preventive health services are central to approaches to public health. *Preventive measures before illness occurs, precautions taken against infectious diseases, awareness of non-infectious diseases, and a risk-based approach to protecting public health* were the most frequently mentioned topics by participants in the “Emphasis on Preventive Healthcare” category.

Participants argue that disease prevention positively affects not only individual health but also social wellbeing. In this context, the code “Taking precautions before disease occurs” emphasizes the importance of early information, screening and counseling services. Academics state that the close contact established by pharmacists with individuals is effective in early warning systems:

“Conceptually, I actually understand preventive health services more. First of all, preventive health services are screening, prevention, and then correct guidance… protecting health before treatment and doing this on a social basis” (S4).

This view points to the necessity of implementing preventive health services with a systematic approach and shows that pharmacists can play an active role in this process. Measures taken against infectious diseases are also considered an important component of preventive health services. Participants state that the widespread use of practices such as screening and vaccination is critical for protecting public health in epidemic situations:

“When it comes to public health issues, we can divide it into several different areas, especially now. I think it is important in preventive health measures. For example, infectious diseases … ” (S9).

#### Individual and social awareness elements

Individual and social awareness plays a decisive role in the success of public health services. In the category of “Individual and Social Awareness Elements,” which stands out among the categories related to perceptions and definitional approaches to public health, the following topics were frequently discussed by the participants: *The need for raising public awareness, the importance of individuals acquiring skills and knowledge, the impact of access to information on public health, and the guiding influence of community pharmacists on individuals.*

Participants state that the effectiveness of protective and preventive measures decreases in environments where the level of public awareness is low. In this context, the code “Need for public awareness” emphasizes the need to increase health literacy:

“When it comes to public health, of course, infectious diseases come to my mind. Personal precautions, measures, how to take them, come to mind. I think that precautions, especially preventive health measures, are more important in terms of public health. I think that society should be made aware. In terms of protection” (S8).

This view shows that pharmacists have the potential to increase public awareness by taking an active role in information processes. The impact of access to information on public health is also discussed. Participants state that ensuring that individuals have access to accurate resources is critical in preventing misinformation:

“The first stage is personal, that is, people treating themselves. Unfortunately, they do this not with the information they get from the right sources, but with hearsay or television approaches” (S7).

This quote reveals that information pollution negatively affects public health practices and that pharmacists can undertake the task of facilitating access to reliable information sources.

The guiding influence of pharmacists on individuals is becoming increasingly important. Participants argue that the easy accessibility of pharmacies provides an advantage for individuals to enter the health system:

“The potential role of pharmacists in public health in Türkiye is not sufficiently evaluated. However, with the right training, empowerment and support, this situation can change. Pharmacists are among the health professionals who interact most with society, and this advantage can be used more strategically to improve public health” (S1).

This statement shows that pharmacists can act as a bridge in public health services and that this role should be developed.

### Conceptual and descriptive framework

In the participants’ opinions, public health is defined as a multidimensional field that brings together different disciplines and encompasses both individual and community health. This structure, in which individuals, institutions, and health professionals interact, reveals the breadth of the field and the need for a holistic approach. In this category, the following topics stood out in the participants’ opinions: *The perception of public health as a multidimensional field, the inclusion of psychological health within public health, the definition of public health as a multidisciplinary structure, and the diversity of practices included within the scope of public health.*

Public health is defined by academics as a multidimensional and multidisciplinary field. Participants state that this concept goes beyond individual health and also includes improving social conditions:

“For me, public health means all kinds of efforts to protect, improve and prevent the general health of the society. Not only individual health, but also improving health conditions at the societal level and providing equal access to health services are part of public health” (S1).

This quote shows that public health has an inclusive framework and that the principle of equality has an important place in this field. The inclusion of psychological health in public health is also noteworthy. Participants argue that addressing physical and mental health in a balanced way increases social wellbeing:

“The fact that the society is actually healthy. We are talking about physical health, but I think psychological health is also included in this… In terms of protection… If precautions are taken before a disease occurs, its spread will be prevented” (S8).

This view reveals that public health is not limited to physical diseases only, and the psychological dimension is also becoming increasingly important.

The multidisciplinary structure of public health requires cooperation between different professional groups:

“I do not think it's enough. In fact, there was a system change in our pharmacy education… Actually, I think public health should be a part of all courses. As I said earlier, public health is a very multidisciplinary field. It is a field of science that feeds from all fields” (S8).

This quote shows that public health education should be enriched with an interdisciplinary approach and that this approach can increase the effectiveness of practices.

### Contact points of public health and community pharmacy

The points where public health services intersect with pharmaceutical practice are becoming increasingly visible. Interviews highlight that pharmacies are no longer merely places for obtaining medication; they are now taking on critical roles in counseling, guidance, and preventive health practices. It is understood that community pharmacy, particularly through its direct contact with individuals, contributes significantly to the healthcare system. In daily practice, the function of pharmacists in the field of public health is less about diagnosis and more about forming the first link in the chain of referral, information, and monitoring mechanisms, thus alleviating the systemic burden. Participants’ opinions on the points of contact between public health and community pharmacy focused on topics such as first aid, immunization, and health counseling; contributions to public health through pharmacy consultations; and the role of community pharmacists in the context of chronic disease monitoring and medication management.

“Of course, since I am an academic in this field, I look at it from a more idealistic point of view. But let's think about it on a daily basis, worldwide… The public health services that a community pharmacist or, as it is now called, a community pharmacist can provide in their daily practice can actually be preventive and prophylactic, as I mentioned earlier. Plus, they can also support some issues in terms of treatment” (S10).

This statement shows that pharmacists play a multifaceted role in public health services and that this role is gradually expanding. Pharmacy consultancy is considered an important tool in contributing to public health. Participants state that pharmacies support the health system with services such as vaccination, screening and information:

“For example, we know that pharmacists in pharmacies in the United States actively participate in vaccination campaigns… So, making pharmacies like vaccination centers can be given as an example of public health” (S10).

This quote suggests that pharmacies have become a part of public health services in international examples and that this model is also applicable in Türkiye.

## Discussion

Our study shows that pharmacy academics perceive public health as a preventive, multidimensional, and multi-stakeholder field and view community pharmacies as underutilised public health assets. The findings suggest that pharmacists’ accessibility could be used more effectively through stronger public health education and formal policies recognising their preventive and counselling roles. Recent studies similarly demonstrate pharmacists’ contributions to vaccination, screening, chronic disease monitoring, and other preventive interventions [14–16, 18–20].

### Conceptual framework and preventive role of public health

Participants’ emphasis on preventing disease before it occurs is consistent with the broader public health focus on organised efforts to protect population health [17]. Their accounts position pharmacists within a shared-responsibility model involving health professionals, institutions, government, and individuals. Evidence from other settings also shows increasing pharmacist involvement in preventive services, including vaccination [21]. Pharmacies’ accessibility and repeated contact with individuals therefore create opportunities for early intervention, counselling, and appropriate referral.

### The position of community pharmacy in public health

Participants described pharmacies as counselling and monitoring settings beyond medicine supply, particularly highlighting chronic disease monitoring, medication management, screening, vaccination, and health counselling [22-29]. This is consistent with literature describing expanding pharmacy roles in primary healthcare and contributions to chronic disease management through education and follow-up [22–24]. Such functions may support health systems by improving access to preventive advice and directing individuals to appropriate services.

### Education and health literacy axis

Health literacy emerged as a central factor affecting the effectiveness of public health services. Participants linked low awareness and reliance on unreliable information with reduced effectiveness of preventive interventions, consistent with evidence of health-literacy challenges in Türkiye [25]. Community pharmacists may help bridge this gap because of their accessibility and capacity to provide reliable, repeated counselling [26-29]. However, participants stressed that this potential depends on strengthening public health competencies within pharmacy education. Public health content should therefore be more clearly integrated into undergraduate curricula and supported through postgraduate and continuing professional development.

### Contributions and limitations of the study

This study contributes an academic perspective on pharmacists’ public health roles in Türkiye and provides qualitative insight into prevention, health literacy, education, and community pharmacy’s position within the health system. The findings may inform educational reforms and policies that formally recognise pharmacists as contributors to preventive services and population health.

Several limitations should be acknowledged. Participants were pharmacy academics from Faculties of Pharmacy in Istanbul, which may limit transferability to other regions of Türkiye. Community pharmacists and other healthcare professionals were not included, so the findings predominantly reflect an academic perspective. Future studies should include geographically diverse participants and additional professional groups and examine how proposed roles can be operationalised and evaluated in practice.

## Data Availability

All data generated or analysed during this study are included in this article. Further enquiries can be directed to the corresponding author.
